# Removing Mn, Cu and Fe from Real Wastewaters with Macrophytes: Reviewing the Relationship between Environmental Factors and Plants’ Uptake Capacity

**DOI:** 10.3390/toxics11020158

**Published:** 2023-02-07

**Authors:** Eder Carlos Lopes Coimbra, Alisson Carraro Borges

**Affiliations:** Department of Agricultural Engineering, Federal University of Viçosa, Viçosa 36570-900, Brazil

**Keywords:** toxicity, phytoextraction, biotope, eco-friendly technology, full scale

## Abstract

Heavy metal pollution creates environmental health concerns. Among these, iron (Fe), copper (Cu) and manganese (Mn) are commonly found in aquatic environments due to the release of wastewaters. Phytoremediation in hydroponics uses macrophytes to treat contaminated environments, and this is influenced by environmental factors. However, the relationship between these factors and the removal of Fe, Cu and Mn by macrophytes is not known. Therefore, a meta-analysis serves to determine the correlations between environmental factors and the removal of these metals in real wastewater by macrophytes, as well as to identify the role of different aquatic forms of macrophytes in phytoremediation. Emergent macrophytes had higher concentrations of manganese in their tissues, and higher bioconcentrations factor of iron and manganese than floating plants. Regardless of the biotope, higher concentrations of Fe and Cu decreased the ability of plants to bioconcentrate them. The correlations among exposure time, pH, dissolved oxygen, nitrogen, phosphorus, photoperiod and metal phytoremediation by plants were also found. It can be concluded that the emergent macrophytes showed better performance in terms of the removal of Fe, Cu and Mn, and that the significant correlations between environmental factors and removal vary with the type of metal and the environmental factor analyzed.

## 1. Introduction

Urbanization, industrialization and increased extension of agricultural practices have been proportional to the increase in surface and/or groundwater pollution through the release of wastewater [1]. Heavy metals (HMs) are of great concern to human and natural health, as they are nonbiodegradable, bioaccumulative and persistent pollutants in nature [2,3,4,5]. HMs are released into the environment through natural factors (e.g., rock weathering), but mainly through domestic and industrial activities such as the mining, electroplating, metallurgy, textiles, battery manufacturing, tanneries, oil refining, paint manufacturing, pesticides, pulp and paper, printing and photographic industries [6]. 

Iron (Fe), copper (Cu) and manganese (Mn) are metals commonly found in aquatic environments. In proper concentrations, these metals play an essential role in plant metabolism [7,8,9,10]. However, in inappropriate concentrations in the environment, these metals cause imbalances in metabolism and toxicity not only in plants but also in animals. For instance, studies that evaluated the metal average concentrations between 1972 and 2019 showed that concentrations of these three metals in rivers and lakes across the planet have more than doubled over the decades and are at levels well above the limits established by the World Health Organization (WHO) and by the United States Environmental Protection Agency (USEPA) [11,12]. As there is no consensus in the literature on the definition of the term “heavy metals” [13], here, we follow the recommendation of Appenroth (2010) [14], who stated that for plant sciences, the term “heavy metal” refers to those belonging to the transition metals, as is the case for Fe, Cu and Mn.

Some heavy metals, as they are not part of metabolism, are non-essential in living beings (e.g., Cr, Pb, As, and Hg) [15]. Thus, in trace concentrations and under suitable conditions, they cause toxicity to aquatic organisms or humans. Therefore, they are cause for concern [16] and should be studied regarding their presence in the environment and their biological effects. However, due anthropogenic activities (industrial, domestic or agro-industrial), other metals are released in large quantities into the environment through wastewater. These metals, such as Fe, Cu, Mn, etc., are usually essential for living beings [17], although needed in microquantity. Nevertheless, in high concentrations, they are toxic to the natural environment (e.g., to aquatic biota) and, due to their bioaccumulative nature, they can biomagnify through the food web and be toxic to humans.

For instance, environmental and human toxicities have already been described for Fe, Cu and Mn. Iron accumulation in humans can cause hemochromatosis (an autosomal recessive disease) [18]. In the aquatic environment, acute toxicity values (capable of causing death) of 6.7 mg/L have been reported in *Daphnia magna* [19]. Manganese can produce neurological problems in humans [20] and it has been shown that when inhaled by rats, it can cause damage to astrocytes in their central nervous system [21]. Copper has been reported in fish species in an acute toxicity value of 14.61 µg/L (*Ptychocheilus oregonensis*) and chronic value (when it affects the organism’s ability to reproduce) of 5.92 µg/L (*Oncorhynchus tshawytscha*) [22]. Thus, given the environmental importance of metals that, although essential to living beings, can be toxic in large quantities, we review the presence in the wastewater of and discuss the ability to remove three of these metals (Fe, Cu and Mn) from wastewater.

Conventional technologies usually used in the removal of HMs include chemical precipitation, ion exchange, adsorption, membrane separation, coagulation–flocculation, photocatalytic degradation, flotation and electrochemical processes, which, though efficient, have disadvantages because of their high cost of implementation/operation and because they generate nontoxic by-products [23]. It is, therefore, necessary to use technologies that are cleaner, less expensive and efficient in removing these pollutants.

Given this context, phytoremediation technology has become a viable and sustainable alternative, with recognized efficiency in the removal of aquatic pollutants, and with low implementation, operation and maintenance costs [4,24]. In addition, there is the possibility of recovering resources through the use of plant biomass in the production of energy or fertilizer [25]. It is a technology in which plants are used to remove, detoxify or immobilize contaminants from different environmental matrices, such as in contaminated water, soil or sediments [26]. 

In hydroponic phytoremediation, the root system or even the fronds of aquatic plants (macrophytes), in association with microorganisms, function as natural filters when they have direct contact with contaminated water [27,28,29]. In these systems, the macrophytes used in phytoremediation are classified by their aquatic structural habitat (biotope) or the aquatic form of the macrophyte, which can be floating, emergent or submerged [30].

However, because of the concentration of contaminants in real wastewater, the occurrence of phytotoxicity can impede the phytoremediation of wastewater [31]. Despite this, the combination of the type of plant and environmental factors such as pH, photoperiod, nitrogen (N), phosphorus (P), dissolved oxygen (DO) and exposure time, which are recognized as promoting the growth and development of plants [32,33], make such species able to better cope with these adverse situations, performing better in the removal of HMs. However, there are no reports of a systematic understanding of the existing relationship between the environmental factors and the removal of Fe, Cu and Mn by macrophytes.

A meta-analysis is an essential tool that makes it possible to aggregate information and discussions through a statistical analysis of the results from independent primary studies, providing support for conclusions about a studied phenomenon, with the capacity to contrast and identify patterns or sources of disagreement [34]. There are few reports of this type of analysis in the area of phytoremediation of aquatic environments contaminated by metals [35], as contaminated soils are studied more often [36,37,38,39,40]. Furthermore, to best of our knowledge, there has been no study on the relationship of the environmental factors mentioned above and the removal of Fe, Cu and Mn from real wastewaters through phytoremediation by macrophytes.

Thus, the main objective of this study was to evaluate, through a meta-analysis, the relationship between the environmental factors and the capacity of macrophytes to remove and absorb Fe, Cu and Mn. The hypothesis was that the conditions of the physical and/or wastewater would influence the removal of these HMs from the wastewater and the absorption capacity of these plants, but in different ways, depending on the type of metal and the plants’ aquatic habitat. For this, the specific objectives were to verify (i) the existing correlations between the conditions of the physical environment (photoperiod, exposure time) and wastewater (metal concentration in the wastewater ([HM]_wastewater_) (pH, DO, N and P) on the capacity of different macrophyte types to absorb Fe, Cu and Mn, and (ii) whether the absorption of these three metals was different between floating macrophytes and those in emergent habitats. 

## 2. Materials and Methods

### 2.1. Literature Search and Data Selection

English-language publications were collected by searching the Scopus “http://www.scopus.com (accessed on 11 July 2022)” and Web of Science “http://apps.webofknowledge.com/ (accessed on 11 July 2022)” databases from January 1990 to 2022 (retrieved on 11 July 2022) to identify studies that had evaluated the use of macrophytes in the hydroponic phytoremediation of waters contaminated by the metals Fe, Cu and Mn. For this purpose, the following search terms were used: “macrophytes”, “phytoremediation”, “phytoextraction”, “heavy metals”, “metals”, “iron”, “Fe”, “copper”, “Cu”, “manganese”, “Mn”, “effluent”, “wastewater” and “wastewater treatment”. 

The search string was (“macrophytes” AND (“phytoremediation” OR “phytoextraction”) AND (“heavy metals”) OR (“metals”) OR (“Fe”) OR (“iron”) OR (“Cu”) OR (“copper”) OR (“Mn”) OR (“manganese”) AND (“effluent”) OR (“wastewater”) OR (“wastewater treatment”)) from the search field TOPIC (Web of Science) or Title-ABS-Key (Scopus). Here, reviews, editorials, conference proceedings, books and book chapters were disregarded for further analysis.

The authors conducted a systematic literature review of the phytoremediation of real wastewater that involved removing Fe, Cu and Mn. The PRISMA methodology was used to identify the articles. It should be noted that prior screening of the articles (title/abstract screening) was performed using the StArt tool [41]. The screening of articles by title/abstract was carried out in order to exclude articles that did not deal with phytoremediation in hydroponic systems, as well as overlapping (duplicates) or irrelevant articles. After that, all articles were read in order to select those that met the established criteria.

The selection of publications for the meta-analysis included the following criteria: (i) studies that evaluated the removal of Fe, Cu and/or Mn through phytoremediation of real wastewaters by macrophytes (submerged, emergent or floating) and (ii) studies that reported the concentrations of these metals in the wastewater (mg L^−1^) and the tissues of aquatic plants ([HM]_plant_, mg kg^−1^). 

Furthermore, we excluded studies that involved the biomonitoring of streams or lakes; those that did not provide or in which it was not possible to calculate the bioconcentration factor (BCF); those that studied, in association, two or more macrophytes in the same hydroponic tank; and those that used nonhydroponic constructed wetlands (CWs). The information extracted from the studies were BCF, [HM]_plant_, percentage of metals removed from the water (%R), pH, [HM]_wastewater_, N, P, photoperiod, DO and exposure time.

### 2.2. Meta-Analysis

For the analysis of data integration, it was assumed that the studies conducted in different locations with different plant growth habits were independent [35]. Studies that tested more than one species or type of wastewater but were not dependent were included in the analyses. Macrophytes were categorized into biotopes (emergent, submerged and floating) and into different families [42] to determine differences among them in the removal of Fe, Cu and Mn from the real wastewater. For the studies in which no BCF values were provided, these were determined according to [2] as shown in Equation (1).

(1) $$\mathrm{BCF}=\frac{{\mathrm{Metal}\;\mathrm{concentration}\;\mathrm{in}\;\mathrm{the}\;\mathrm{plant}\;\mathrm{tissues}\;(\mathrm{mg}\;\mathrm{kg}}^{-1})}{{\mathrm{Metal}\;\mathrm{concentration}\;\mathrm{in}\;\mathrm{the}\;\mathrm{wastewater}\;(\mathrm{mg}\;L}^{-1})}$$

Moreover, for studies that provided values of BCF for the roots (BCF_root_) and the aerial parts (BCF_aerial_), the sum of BCF_root_ and BCF_aerial_ was considered to be the plant’s BCF [43]. When this was the case, stem and leaf parts were considered to be aerial parts of the plant. Nitrogen (N) and phosphorus (P) concentrations reported in different formats (nitrate, ammonium, ammoniacal nitrogen and phosphate) were converted to their respective molar concentrations of N (mmol N L^−1^) or P (mmol P L^−1^). The removals of metals from the wastewater, when not provided, were calculated according to Equation (2):

(2) $$\%R=\frac{C_{i}{-\;C}_{f\;}}{C_{i}}\times 100$$

where C_i_ is the initial metal concentration in the wastewater, in mg L^−1^; C_f_ is the final metal concentration in the wastewater, in mg L^−1^; and %R represents the total removal of metals from the wastewater.

### 2.3. Statistical Data Analysis

To eliminate the differences caused by the experimental conditions, the species, the initial metal concentration and other sources of variation, the BCF and [HM]_plant_ data were transformed to a Neperian logarithm (ln). This transformation allowed the observed variability to be compressed but did not change the relationship between the observed points [35]. The data of ln(BCF), ln[HM]_plant_ and the percentage of metal removed (%R) were correlated with the environmental factors ([HM]_wastewater_, pH, exposure time/experimental duration, N, P, DO and photoperiod) according to Spearman’s monotonic correlations (r_s_) at the 5% significance level [44,45].

The normality of the distribution (Shapiro–Wilk, α = 0.05) was checked beforehand. Correlations were examined when there were at least seven pairs of interactions and were always for the entire plant regardless of the type of structural habitat [46]. Fisher’s transformation Z test (α = 0.05) was used to compare the rs coefficients when they were significant [47]. 

The species were categorized into emergent and floating biotopes, and differences in their Fe, Cu, and Mn uptake were verified. For this purpose, comparisons were made between the two biotopes in terms of the uptake, ln(BCF) and ln[HM]_plant_ for the same metal according to the nonparametric Mann–Whitney test (α = 0.05). Data in graphs and figures were extracted using Image J software “https://imagej.nih.gov/ij/ (accessed on 11 July 2022)”, and statistical analyses were performed with GraphPrism v.6 (GraphPad Software, San Diego, CA, USA).

## 3. Results

### 3.1. Literature Search

In total, 225 publications were selected from Scopus (72 publications) and Web of Science (153 publications). Of this total, 13 studies met the selection criteria (Section 2.1). All studies were concentrated in Asia, with most conducted in India (76.9%), followed by Pakistan, Malaysia and Japan, with one study each (Table 1).

The wastewaters studied were domestic (municipal and graywater) and industrial (pulp and paper, paper, palm oil, mining and metallurgy). In addition, 13 families (Table 1) and 16 species divided into the emergent (8), floating (7) and submerged (1) biotopes were reported in the selected studies. 

The species *Lemna minor* (Lemnaceae) and *Pistia stratiotes* (Araceae) were the most frequently studied, with four studies each. In addition, the concentrations of the three metals in the wastewaters ranged across 0.104–22.91, 0.032–4.64 and 0.007–230 mg L^−1^ for iron, copper and manganese, respectively. Some studies performed phytoremediation in more than one type of wastewater but independently [43,48] (Table 1).

### 3.2. Accumulation of Fe, Cu, and Mn in Plant Tissues

Overall, the bioconcentration factors, expressed as ln(BCF), and the concentrations of metals in plant tissues, expressed as ln[HM]_plant_, presented great variability among the macrophytes and for each type of metal analyzed. Except for the emergent macrophytes that took up manganese, for which the coefficient of variation (CV) was 5% and 33% for ln[HM]_plant_ and ln(BCF), respectively, all the others showed high CVs, some higher than 300%, as in the case of ln[HM]_plant_ for the floating macrophytes used for phytoremediation of copper-contaminated waters. Despite this, all statistical analyses were performed with all data, without excluding the outliers.

**Figure 1:**
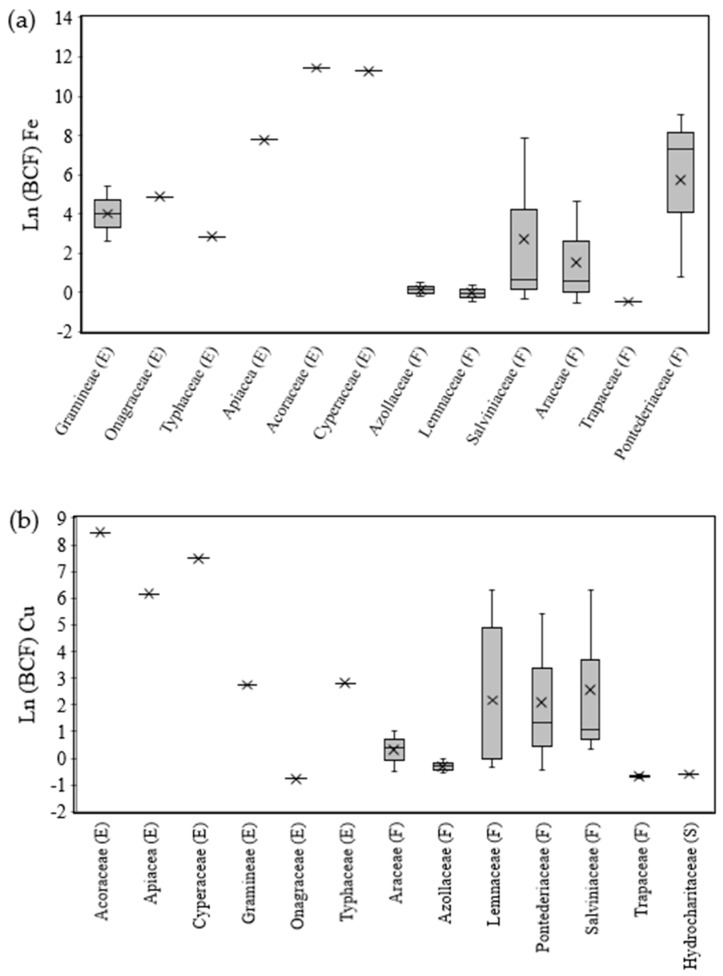

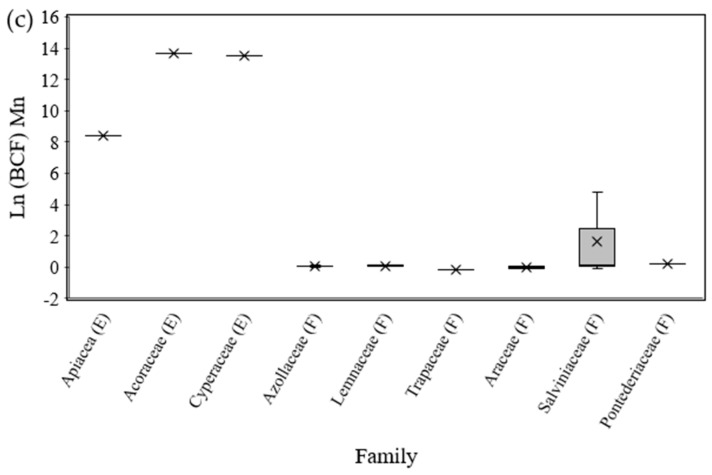
Boxplot of bioconcentration factors (BCF), expressed as ln, for iron (Fe) (**a**), copper (Cu) (**b**) and manganese (Mn) (**c**). The symbol “X” represents the mean. (E) Emergent families of macrophyte; (F) floating families of macrophyte; (S) submerged families of macrophyte.

The most and least frequently reported metals were copper and manganese, respectively (Table 1). The ln(BCF) data of the selected macrophyte species varied according to the type of metal and the type of family used in the study (Figure 1a,b). In general, the emergent macrophyte families showed higher ln(BCF) values for all three metals, although some species were reported only once. There were no significant differences in ln(BCF) and ln[HM]_plant_ values among roots, aerial parts (leaves/stems) and whole plants for each metal, according to the Kruskal–Wallis nonparametric test at 5% significance (data not shown).

The ln(BCF) data for iron, copper and manganese showed, regardless of the family type or biotope, means (µ) of 3.462 (*n* = 21, median = 2.756, minimum = −0.555, maximum = 9.998), 2.008 (*n* = 25, median = 0.380, minimum = −0.777, maximum = 8.455) and 2.122 (*n* = 14, median = 0.113, minimum = −0.146, maximum = 8.421), respectively.

For the differences in the three metals among families, ln(BCF) values higher than 6.9 (BCF > 1000) were observed for the families Acoraceae (*Acorus gramineus*), Cyperaceae (*Cyperus alternifolius* L.) and Apiaceae (*Centella asiatica*), which were reported only once, in works that studied the removal of these metals in wastewaters from the pulp and paper industry [51] or metallurgy [53] (Figure 1a–c). On the other hand, among the floating species and for the metal iron, the Pontederiaceae family, represented by *Eichhornia crassipes*, although they presented ln(BCF) values below 6.9 on average (Figure 1a), there was a case in which this species had ln(BCF) > 6.9 when used for the phytoremediation of graywater [43].

**Figure 2:**
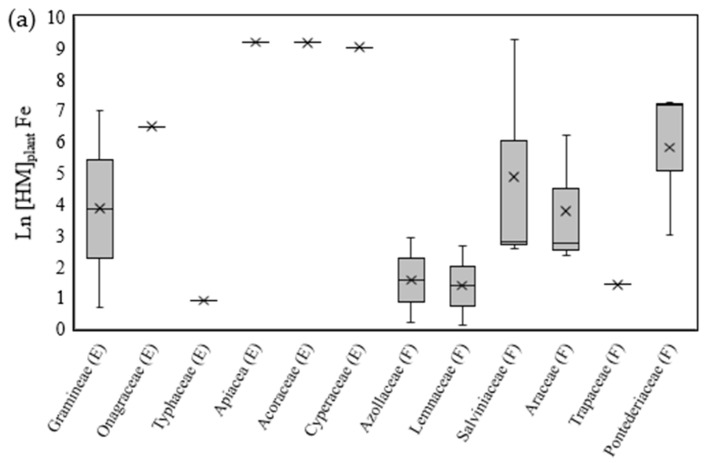

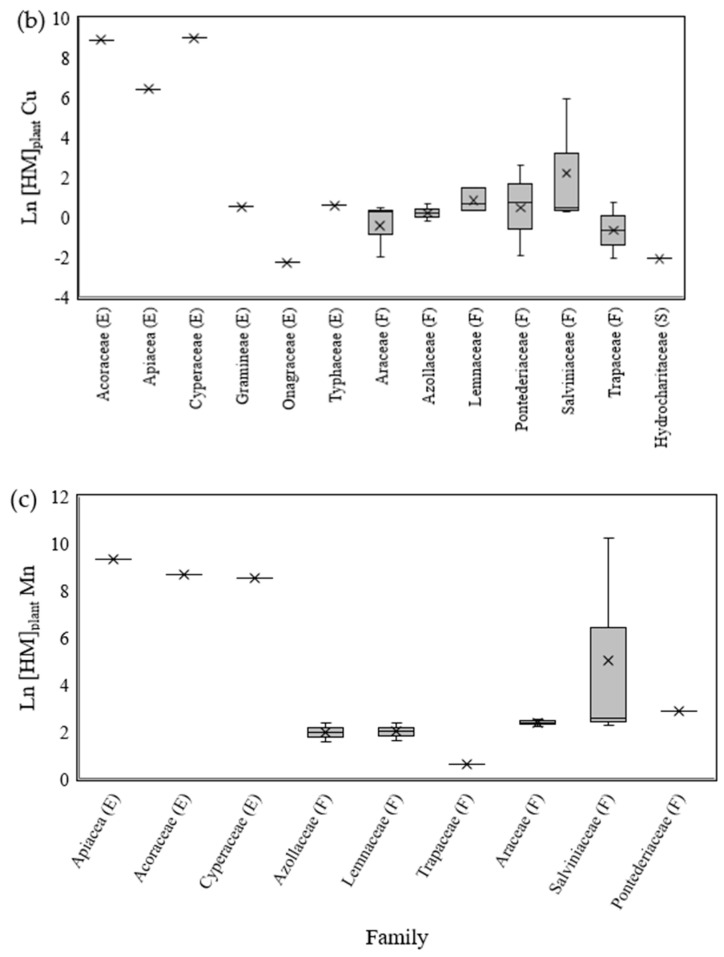
Boxplot of the concentrations of HMs in plant tissues, expressed in ln, for iron (**a**), copper (**b**) and manganese (**c**). The symbol “X” represents the average. (E) Emergent families of macrophyte. (F) Floating families of macrophyte. (S) Submerged families of macrophyte.

The concentrations of metals in plant tissues, expressed as ln[HM]_plant_, also varied with the type of metal and the type of family used in the study (Figure 2a,b). Similar to ln(BCF), emergent macrophytes showed higher ln[HM]_plant_ values for all three metals, although some were reported only once.

The concentrations of metals in plants, ln[HM]_plant_, for iron, copper and manganese showed, regardless of the family type or biotope, means (µ) of 4.44 (*n* = 21, median = 2.938, minimum = 0.12, maximum = 9.253), 1.268 (*n* = 25, median = 0.546, minimum = −2.246, maximum = 8.997) and 4.134 (*n* = 14, median = 2.453, minimum = 0.624, maximum = 10.240), respectively.

For the differences among families, there were concentrations below 1 only for copper (ln[HM]_plant_ < 0) and mainly among the floating macrophytes (Figure 2b). Again, the highest ln[HM]_plant_ values occurred for the families Acoraceae (*Acorus gramineus*), Cyperaceae (*Cyperus alternifolius* L.) and Apiaceae (*Centella asiatica*) in studies on the removal of these metals in wastewaters from the pulp and paper industry [51] or metallurgy [53] (Figure 2a–c).

The species were subgrouped into floating and emergent biotopes and showed differences between the two biotopes in the ranked mean comparison values (mean rank) of ln(BCF) and ln[HM]_plant_ for metal uptake according to the Mann–Whitney test at 5% significance (Table 2). The submerged macrophyte *Hydrilla verticillata* (Hydrocharitaceae) was not considered because it was reported in only one study (ln(BCF): −0.598; ln[HM]_plant_: −2.068) [52].

To sum up, emergent macrophytes had the highest ranked mean ln(BCF) values relative to floating macrophytes, with significant differences (*p* ≤ *0.05*) between the two biotopes for iron and manganese (Table 2). Furthermore, the U = 0 statistic between floating and emergent species in the ln(BCF) value for manganese indicates that all ln(BCF) values for the emergent macrophytes were greater than those for the floating ones. For ln[HM]_plant_, the emergent species also showed the highest mean ranks compared with the floating species, but the emergent species tended to have significantly higher ln[HM]_plant_ values (*p* ≤ *0.05*) only for manganese when compared with the floating species.

### 3.3. Correlations between Environmental Factors and the Removal and Concentration of Metals in Plant Tissues

The conditions of the physical and wastewater environment (environmental factors), reported by the parameters [HM]_wastewater_, pH, exposure time (t), N, P and DO concentrations, and the photoperiod were correlated with the responses ln(BCF), ln[HM]_plant_ and %R (Table 3). The exposure time parameter obtained more significant Spearman correlation coefficients (r_s_) (*p* ≤ *0.05*), with positive values of 0.727 (ln(BCF)) and 0.649 (ln[HM]_plant_) for iron and 0.462 (ln[HM]_plant_) for copper. On the other hand, for the photoperiod, there was a strong negative correlation with at least one of the three measured responses for all three metals, with rs values between −0.437 and −0.760 for Fe, Cu and Mn (Table 3). Spearman coefficients, positive or negative, are considered to be strongly correlated or directional when they are between ±0.5 and ±1.0 [60].

Among the metals, there were more significant rs coefficients (*p* ≤ *0.05*) for copper, which correlated positively or negatively with all factors except for nitrogen in terms of ln(BCF), ln[HM]_plant_ or %R. Furthermore, for iron, only the exposure time and the concentration of the metal in the wastewater influenced the values of %R, ln(BCF) and ln[HM]_plants_. For manganese, only pH, exposure time and photoperiod correlated significantly with at least one of the measured responses ln(BCF), ln[HM]_plant_ or %R. In this case, the smaller number of environmental factors with significant correlations for Fe and especially for Mn can be attributed to the smaller sample sizes [36,61].

For the macronutrients nitrogen and phosphorus, only the concentration of phosphorus and copper correlated (*p* ≤ *0.05*), with the metal concentration in plant tissues showing positive rs values of 0.601 for ln(BCF) and 0.842 for ln[HM]_plant_. The same behavior was obtained for dissolved oxygen, with coefficients of 0.506 for ln(BCF) and 0.526 for the correlation with ln[HM]_plant_. There were no correlations between the parameter of nitrogen concentration with ln(BCF), ln[HM]_plant_ or %R for any of the metals.

A strong positive correlation (*p* ≤ *0.05*) was obtained between [HM]_wastewater_ and %R for iron, with r_s_ = 0.876. On the other hand, the strongest negative correlation (*p* ≤ *0.05*) was obtained between photoperiod and ln(BCF) for manganese with r_s_ = −0.760. Comparisons of the order of magnitude between the significant coefficients indicated differences between the coefficients of photoperiod (−0.437) and [HM]_wastewater_ (0.876) and %R for the metal Fe (*p* ≤ *0.05*). On the other hand, for ln[HM]_plant_ of the metal Cu, there were differences (*p* ≤ *0.05*) between the correlation coefficients of DO (0.526), photoperiod (−0.627) and time (0.462) (Table 3).

## 4. Discussion

### 4.1. Accumulation of Fe, Cu and Mn in Plant Tissues

The concentration of metals in plants, expressed as ln[HM]_plant_, indicates how much of the metal was present in the plant tissue at harvest time [36]. However, this concentration alone is not enough to determine the real capacity of plants to absorb metals into their tissues [62]. A plant can have a high concentration of the metal in its tissues but not necessarily have a high capacity to remove it from the wastewater during the exposure time. In this sense, the bioconcentration factor, expressed here as ln(BCF), best represents the relative absorption of the plant for concentrating metal from the wastewater [29].

Overall, among the families, emergent macrophytes showed the highest mean values of ln(BCF) and ln[HM]_plant_ for all three metals, as shown in Figure 1a–c and Figure 2a–c. This biotope, however, was comparatively less frequently reported than the floating biotope. In hydroponic phytoremediation systems, floating macrophytes are often used, given their greater ease of management and operation [4].

However, the individual performance of a macrophyte in the phytoremediation of contaminated water may vary depending on the type of wastewater and the environmental conditions of the experiments [29]. When we grouped the families into the emergent and floating biotopes to compare their performance (Table 2), the mean values of ln(BCF) for Fe and Mn and ln[HM]plant for Mn of the emergent species were higher than those of the floating ones, indicating that the former were more effective in the uptake of Fe and Mn compared with the latter group for the phytoremediation of real wastewater, but this was not the case for copper. Root architecture is an important factor that improves water quality [63] and plants with more roots can assist the development of mycorrhizal fungi, bacteria or algae. These organisms occur in symbiosis with macrophytes’ roots and can improve the plants’ uptake of pollutants [64]. Thus, as the calculated BCF value did not distinguish between the accumulation in the roots and in the aerial parts (Section 2.2), it is possible that the roots of the selected emerging macrophytes contributed to the higher value of ln (BCF) than floating macrophytes. Macrophytes from emergent habitats are recognized for having voluminous roots.

Furthermore, among the metals, copper and manganese presented the lowest mobility from the wastewater to the plant tissues, with the lowest mean values of ln(BCF). Except for the macrophytes with emergent habits, all floating macrophytes presented ln(BCF) values lower than 6.9, indicating the low uptake capacity of these two metals by these macrophytes. An ln(BCF) value of less than 6.9, which corresponds to a BCF value of <1000, indicates the low capacity of plants to bioconcentrate an element in their tissues [29].

It can be argued that the concentration of copper in plant tissues depends on the type of plant, its stage of development and environmental factors [65]. Furthermore, in wastewater, copper ionizes and forms complexes with the organic and inorganic matter present in the wastewater, which may affect its bioavailability to plants [66]. Manganese is poorly mobile in plants and its oxidized form (Mn^2+^), in which plants require it, is sensitively dependent on the optimal supply regulated by Mn transporters in the rhizosphere [67]. In addition, although it has seldom been studied in aquatic systems, in multi-element environments, there may be competition for active binding sites on the macrophytes, increasing or decreasing the uptake of some metals relative to others [9,68].

Finally, it should be noted that, although phytoremediation is a promising method for controlling pollution, the biomass produced by the remediation process must be managed; otherwise, it will eventually return to the environment and cause secondary pollution. In the literature, there are some routes for the disposal of biomass after phytoremediation. In all of them, volume reduction is essential for proper recovery of heavy metals.

For instance, in the incineration (e.g., combustion process), biomass can be used for energy generation and the remaining ash (which contains heavy metals) can be used as phyto(bio)ore [69] or discarded under controlled conditions. Another solution is composting, through which, although it reduces the volume of biomass, high amounts of heavy metals or the presence of certain HMs make the agricultural use of the compost unfeasible [70]. In this sense, stabilization/inertization techniques have been used, such as mixing with other materials or adding lime to reduce the leachability of metals [71]. Regarding leaching, this can be obtained by treating the compacted biomass with solvents in which the metallic components are extracted from the leachate and disposed of under suitable conditions [72]. Therefore, the fate of these plants after their use in wastewater treatment should be part of the study for the implementation of the phytoremediation technique.

### 4.2. Correlations between Environmental Factors and the Removal and Concentration of Metals in Plant Tissues

The bioconcentration of Fe and Cu by plants indicates that the phytoextraction of these metals is less effective as their concentrations in the wastewater increase (Table 3). This may occur when, at high concentrations, the metabolic costs to plants of absorbing them are high [36] or as a result of the control that plants have in regulating the levels of metals in their plant cells in situations with high concentrations of heavy metals in the wastewater [73,74]. Furthermore, in the context of exposure to complex environments, such as in real wastewater, plants may expend more energy in counteracting possible oxidative stress than on the uptake of HMs [75,76].

Interestingly, the exposure time showed opposite relationship between the removal of metals from the wastewater and their absorption in plant tissues. For all three metals, the removal (%R) of metals was higher in tests with shorter durations (Table 3). On the other hand, for Fe and Cu, an increase in ln[HM]_wastewater_ or ln(BCF) occurred with longer durations (Table 3). Plants, when exposed to a new adverse condition, may undergo an adaptation time before they begin remediation [29]. In high-toxicity environments, the oxidative stress defense system is triggered until the plants can adapt to the new environment and remediate it [75]. In hydroponic phytoremediation systems, besides the uptake by plants, bacteria and algae, abiotic mechanisms such as binding to suspended solids and precipitation as insoluble compounds are among the main mechanisms of heavy metal removal [26]. Thus, it might be that at the beginning of the tests, the metals are removed (%R) from the wastewater preponderantly through the action of other mechanisms such as rhizobacteria, but not by the plants.

The pH value is one of the most important factors influencing the bioavailability of metals in plants [77], and small changes in this value can cause an increase or decrease in the concentrations by an order of magnitude compared with the surrounding environment [78]. Although the efficiency of metal uptake by plants varies in terms of the ideal pH range, in general, at a higher pH, metal ions form insoluble oxides, such as hydroxides, and plants are unlikely to take them up [35]. On the other hand, at a lower pH, there is an increase in the concentration in the medium and increased availability to the plants [9,78]. In the selected studies, the vast majority of wastewater had neutral to alkaline pH ranges, which are likely to lead to negative correlations between the pH values and plant uptake values. However, there were negative correlations (*p* ≤ *0.05*) between pH and the removal (%R) of Cu and Mn, which indicates that greater removal of these metals occurs at lower pH values.

For manganese, however, the positive correlation (*p* ≤ *0.05*) between pH and ln(BCF) indicated that plants are also able to remove and bioconcentrate this metal in their plant tissues at an alkaline pH. For cationic species, such as Mn^2+^ (plant-available fraction), higher pH values result in lower mobility and, therefore, lower availability to plants. This is because as the pH increases, the hydrolysis of the metal increases, forming insoluble compounds. Moreover, at a higher pH, the electro-negative charge on the surfaces of the variable-charge colloids present in the wastewater also increases, which causes these metals to be retained [76]. However, increased metal availability may occur at higher pH values, which can be attributed to the precipitation of its soluble forms in carbonate fractions, which are mobilized in the acidic rhizosphere zone of the plants [79].

The bioconcentration factor does not distinguish between metal uptake and what can be adsorbed on the plants’ root surface [29], although it is customary to evaluate the absorption capacity of plants using the bioconcentration factor. In this sense, other indices should be considered for a better evaluation of absorption, such as the tolerance index (TI), the adsorption factor (AF) and the translocation factor (TF) [29].

Phosphorus and dissolved oxygen correlated significantly (*p* ≤ *0.05*) with the ln(BCF) and ln[HM]_plant_ of copper. On the other hand, in none of the metals were there any correlations (*p* ≤ *0.05*) of N with the variables of ln(BCF), ln[HM]_plant_ and (%R), which indicates that nitrogen does not seem to affect the removal and/or uptake of Fe, Cu and Mn from real wastewater treated by macrophytes.

Dissolved oxygen, by increasing aeration in the rhizosphere region, plays an essential role in supporting the growth of aerobic bacteria [52]. These bacteria increase the capacity of plants to absorb heavy metals by changing their uptake properties, such as root growth, or by acting to reduce phytotoxicity [76]. Furthermore, it has been shown that aeration of the rhizosphere can induce the formation of more developed aerenchyma and increase the capacity for metal uptake and bioaccumulation [80].

Phosphorus is the second most important macronutrient for plant nutrition and growth [81]. In terrestrial environments, P compounds act as a source of heavy metals for plants either by direct metal adsorption, phosphate anion-induced metal adsorption and desorption or modification of the rhizosphere through acidification and mycorrhizal associations [82]. Copper is associated with its transporters from the surrounding medium to plant tissues, such as the COPT family of proteins, which also participate in the metabolism of phosphate [74,83,84].

Similar to P, N is also a macronutrient and is essential for plant growth [85,86]. In this case, it could have an indirect effect on the removal of metals since it is associated with an increase in plant biomass. Although species with a larger biomass generally have higher concentrations of elements, this does not necessarily imply their better performance in terms of the accumulation and translocation of elements [87]. Studies have suggested that several plant species living in environments that are susceptible to high pollutant loads, such as wetlands, tend to adopt a strategy of tolerance by relying on roots (and rhizomes) as the main accumulating organs, which can store most of the trace elements to protect themselves against the harmful effects of toxic concentrations on photosynthetic organs [88].

For instance, emergent macrophytes in a hydroponic system grow on a mat or raft floating on the surface of the water rather than being rooted in the sediment/substrate. The roots, normally more voluminous and developed, are below the floating mat and provide a large surface area for biofilms to attach. In these systems, the vegetation mat is larger and more consolidated than that of free-floating systems (here called floating macrophytes), which might makes them more able to remove pollutants [89].

The photoperiod is the amount of light in a 24 h daily cycle. In plants, the photoperiod controls responses such as circadian rhythms and light signal detection in the leaves [90]. The uptake and concentration of Fe and Cu are higher in shorter photoperiods, with which strong negative correlations (*p* ≤ *0.05*) were found (Table 3). However, with longer periods of light exposure, there may be changes in the redox state of chloroplasts, which includes responses to environmental stress as one of its functions. Under conditions of high light exposure or high light intensity, there is a need for the acclimatization of the photosynthetic machinery to avoid damage to the antioxidant defense system [91]. Therefore, with the combination of the stressful conditions of the wastewater environment and the longer light period, plants decrease the uptake of metals to avoid such damage.

It was observed in the meta-analysis that the environmental factors ([HM]_wastewater_; pH; exposure time; the concentrations of N, P, and DO; and photoperiod) affected the removal and uptake of Fe, Cu and Mn, but with significant correlations that varied with the type of metal and the parameter analyzed. However, it is important to verify the magnitude of the coefficient to the correlated variable. The magnitude does not depend on the correlation’s sign but on the sample size (n) used for the correlation [47].

For this purpose, comparisons were made between the significant coefficients of the environmental factors with the same correlated variable. In almost all of them, there were no significant differences according to Fisher’s Z-test of transformation (*p* ≤ *0.05*). For example, for the ln(BCF) of iron, there were no differences between the coefficients of time (0.727) and [HM]_wastewater_ (−0.703) regarding the ability of macrophytes to bioconcentrate this metal in their tissues (Table 3). On the other hand, for ln[HM]_plant_ of copper, there were differences between the coefficients of exposure time (0.462) and DO (0.526) even though they were coefficients with strong correlations.

## 5. Conclusions Remarks

In this study, we performed a meta-analysis of research from the past 30 years (1990–2022) on the relationship between factors in the physical and aqueous environments and the removal of Fe, Cu, and Mn from real wastewater through phytoremediation using hydroponically grown floating, emergent and submerged macrophytes. Emergent macrophytes, although less often reported, showed higher mean ln(BCF) values for iron and manganese, and higher ln[HM]_plant_ values for manganese than their floating counterparts. There was only one report of a submergent macrophyte. Thus, research with submerged macrophytes treating wastewater and evaluating Fe, Cu, and Mn should be encouraged in order to also understand the dynamics of removal of these metals by this type of macrophyte.

In addition, Cu and Mn showed less mobility from the wastewater to plant tissues, regardless of the biotope. There were significant correlations between the factors of the physical medium (exposure time and photoperiod) and the wastewater (pH, initial metal concentration in the wastewater, N, P and DO), but in different ways: Regardless of the aquatic form of the macrophytes, higher initial concentrations of Fe and Cu in the effluent decreased the ability of plants to bioconcentrate them in their plant tissues.

(i) Shorter exposure times or test durations increased the removal of Fe, Cu and Mn from the wastewater, but hindered the uptake of Fe and Cu.
(ii) Shorter daylengths increased the plants’ ability to absorb the three metals and remove them from the wastewater.
(iii) Lower pH increased the removal of Cu and Mn from the wastewater, but decreased the uptake of Mn.
(iv) Higher concentrations of P and DO increased the uptake and concentration of Cu in plant tissues.

## Figures and Tables

**Table 1:** Summary of some information extracted from the selected studies to evaluate the Fe, Cu and Mn uptake capacity.

Study	Country	Family	Initial Metal Concentration in the Wastewater (mg L^−1^)			Ref.
			Fe	Cu	Mn	
1	India	Pontederiaceae	0.153–0.975	0.062	__	[43]
2	Pakistan	Lemnaceae	__	0.032–0.062	__	[48]
3	India	Azollaceae, Lemnaceae	22.91	2.04	9.61	[49]
4	India	Araceae, Pontederiaceae, Salviniaceae	9.01	0.56	14.25	[50]
5	India	Apiaceae	4.15	1.32	2.56	[51]
6	India	Araceae, Lemnaceae Pontederiaceae, Rapaceae Onagraceae, Hydrocharitaceae	__	0.23	__	[52]
7	Japan	Acoraceae, Cyperaceae	0.104	0.135	0.007	[53]
8	India	Azollaceae, Lemnaceae	0.762	1.432	4.957	[54]
9	India	Salviniaceae	3.9	0.684	230	[55]
10	Malaysia	Araceae, Gramineae, Onagraceae	5	__	__	[56]
11	India	Trapaceae	6.75	4.64	2.16	[57]
12	India	Gramineae, Typhaceae	0.145	0.11	__	[58]
13	India	Araceae, Salviniaceae	18.21	0.93	8.47	[59]

The symbol (__) indicates that the metal was not mentioned in the referred study.

**Table 2:** Comparison between emergent and floating biotopes in the absorption of Fe, Cu and Mn.

Comparison	ln(BCF)			ln[HM]_plant_		
	Mean Rank	U	*p*-Value	Mean Rank	U	*p*-Value
Floating–Fe vs. Emergent–Fe	8.64	16.0	0.012 *	9.86	33.0	0.247 ^ns^
	15.71			13.29		
Floating–Cu vs. Emergent–Cu	11.06	28.0	0.089 ^ns^	11.39	34.0	0.199 ^ns^
	16.83			15.83		
Floating–Mn vs. Emergent–Mn	6.00	0.0	0.005 *	6.27	3.0	0.038 *
	13.00			12.00		

^ns^ Not significant. * Significant according to the Mann–Whitney test (*p* ≤ *0.05*). N_floating_: 14 (Fe), 18 (Cu) and 11 (Mn). N_emergent_: 7 (Fe), 6 (Cu) and 3 (Mn).

**Table 3:** Spearman’s correlation coefficients (r_s_) for the factors of metal concentration in the wastewater ([HM]wastewater), pH, time, nitrogen, phosphorus, DO (dissolved oxygen) and photoperiod with the ln of the bioconcentration factor (BCF), the metal concentration in the plant ([HM]plant) and the percentage of metal removed (%R) from the wastewater.

Factors	Fe			Cu			Mn		
	ln(BCF)	ln[HM]_plant_	%R	ln(BCF)	ln[HM]_plant_	%R	ln(BCF)	ln[HM]_plant_	%R
[HM]wastewater	**−0.703 ^a^**	−0.184 ^ns^	**0.876 ^a^**	**−0.422 ^a^**	−0.117 ^ns^	0.146 ^ns^	−0.233 ^ns^	0.179 ^ns^	0.366 ^ns^
pH	0.366 ^ns^	0.368 ^ns^	−0.374 ^ns^	−0.212 ^ns^	−0.226 ^ns^	**−0.616 ^a^**	**0.539 ^a^**	0.303 ^ns^	**−0.543 ^a^**
t	**0.727 ^a^**	**0.649**	**−0.759 ^ab^**	0.380 ^ns^	**0.462 ^a^**	**−0.574 ^a^**	0.431 ^ns^	0.526 ^ns^	**−0.759 ^a^**
Nitrogen	0.254 ^ns^	0.485 ^ns^	−0.441 ^ns^	−0.138 ^ns^	0.229 ^ns^	−0.465 ^ns^	__	__	__
Phosphorus	0.036 ^ns^	0.291 ^ns^	0.103 ^ns^	**0.601 ^a^**	**0.842 ^ab^**	0.126 ^ns^	__	__	__
DO	0.127 ^ns^	0.461 ^ns^	−0.438 ^ns^	**0.506 ^a^**	**0.526 ^b^**	0.038 ^ns^	0.410 ^ns^	0.148 ^ns^	−0.48 ^ns^
Photoperiod	0.220 ^ns^	−0.235 ^ns^	**−0.437 ^b^**	**−0.596 ^a^**	**−0.627 ^b^**	0.450 ^ns^	**−0.760 ^a^**	**−0.755**	−0.25 ^ns^

Units: [HM]wastewater and DO (dissolved oxygen), mg L^−1^; t (time of exposure/experiment), days; Phosphorus (P) and nitrogen (N), mmol L^−1^; photoperiod, hours. The significant correlation coefficients between environmental factors (factors) with ln(BCF), ln[HM]_plant_ or %R (*p*-values less than or equal to 0.05) are indicated with a positive or negative sign and emphasized in bold. At the same time, different letters within each column indicate significant differences among the correlation coefficients according to Fisher’s transformation Z-comparison test with *p* ≤ *0.05*. ^ns^, not significant. The symbol (__) indicates that the calculation of r_s_ was not possible (n < 7).

## Data Availability

Not applicable.
